# Consumer and health professional reflections and feedback on the Help After Leaving hospiTal delirium rehabilitation programme

**DOI:** 10.1093/ageing/afag269

**Published:** 2026-09-16

**Authors:** Yonas Tesfaye, Courtney R Davis, Melissa J Hull, Danielle Greaves, Clare Haylock, Sally Johns, Alice Bourke, Tobias Loetscher, Amanda Hutchinson, Stephanie Wong, Ashleigh E Smith, Lisa Kalisch Ellett, Aileen Collier, Monica Cations, Kate E Laver, Hannah A D Keage

**Affiliations:** School of Psychology, College of Education, Behavioural and Social Sciences, Adelaide University, Adelaide, South Australia, Australia; School of Psychology, College of Education, Behavioural and Social Sciences, Adelaide University, Adelaide, South Australia, Australia; Adelaide University Online, Academic Portfolio, Adelaide University, Adelaide, South Australia, Australia; Adelaide University Online, Academic Portfolio, Adelaide University, Adelaide, South Australia, Australia; Northern Adelaide Local Health Network, SA Health, Adelaide, South Australia, Australia; Northern Adelaide Local Health Network, SA Health, Adelaide, South Australia, Australia; Northern Adelaide Local Health Network, SA Health, Adelaide, South Australia, Australia; School of Psychology, College of Education, Behavioural and Social Sciences, Adelaide University, Adelaide, South Australia, Australia; School of Psychology, College of Education, Behavioural and Social Sciences, Adelaide University, Adelaide, South Australia, Australia; College of Human Sciences and Culture, Flinders University, Adelaide, South Australia, Australia; School of Allied Health and Human Performance, College of Health, Alliance for Research in Exercise Nutrition and Activity (ARENA) Research Centre, Adelaide University, Adelaide, South Australia, Australia; School of Pharmacy and Biomedical Sciences, College of Health, Adelaide University, Adelaide, South Australia, Australia; College of Health and Enablement, Research Centre for Palliative Care, Death and Dying, Flinders University, Adelaide, South Australia, Australia; College of Human Sciences and Culture, Flinders University, Adelaide, South Australia, Australia; Caring Futures Institute, College of Nursing and Health Sciences, Flinders University, Adelaide, South Australia, Australia; School of Psychology, College of Education, Behavioural and Social Sciences, Adelaide University, Adelaide, South Australia, Australia

**Keywords:** delirium, rehabilitation, workshop, qualitative research, co-production, older people

## Abstract

**Background:**

Delirium in late life is associated with long-term adverse clinical outcomes. There is no standard rehabilitation pathway following hospitalisation for individuals who experienced delirium. Through co-production we have been developing Help After Leaving HospiTal (HALT), a multicomponent, tailored and scalable intervention designed to prevent cognitive decline in individuals without dementia following in-hospital delirium. This study aimed to gather feedback on the HALT programme’s structure, content, resources and engagement strategies prior to pilot testing from individuals who have treated, cared for or experienced delirium.

**Methods:**

A descriptive qualitative study was undertaken with health professionals (*n* = 12) and consumers (*n* = 4). Five in-person workshops were held in August and September 2025. A semi-structured workshop guide was used to elicit participant reflections and feedback. Audio-recorded data were transcribed verbatim, coded using NVivo and analysed using thematic analysis.

**Results:**

Five themes were generated: (i) personalisation and enhanced support; (ii) beyond involvement, supporting support persons is essential; (iii) be a trusted partner; (iv) overcoming barriers to optimise engagement and (v) comprehensive content and easy to read materials.

**Conclusion:**

Feedback has been used to shape the HALT programme prior to pilot testing by increasing the flexibility of module delivery methods, incorporating information for support persons, ensuring strong linkages with hospitals and overcoming barriers to optimise engagement to ensure the programme meets the needs of its intended users. These insights may also be leveraged by others working on delirium rehabilitation programmes.

## Key points

- Feedback on HALT was gathered through co-production processes to ensure it meets its intended purpose.
- Tailored support, assistance for support persons and strong relationships were deemed critical to the programme’s success.
- Flexible delivery, awareness of programme benefits and motivational interviewing may help address barriers to engagement.
- The modular programme was seen as comprehensive and suitable for older adults who have experienced delirium.

## Introduction

Delirium is a common condition affecting up to 40% of hospitalised older adults [1] and is associated with an increased risk of cognitive impairment (odds ratio/OR = 2.1), functional decline (OR = 2.0), dementia (OR = 5.4), mortality (OR = 2.6), hospital readmission (OR = 1.7) and institutionalisation (OR = 2.8), along with poorer mental health and quality of life following hospital discharge [2]. These adverse outcomes present substantial long-term and costly challenges for patients, support persons and health systems [3, 4]. The cost of delirium among older adults in the USA in 2020 showed that delirium adds $34 828 USD for those with Alzheimer’s disease related dementias (ADRD) and $21 608 USD for those without ADRD to 1-year healthcare costs, compared to those without delirium [5]. Despite these impacts, there is no standard rehabilitation pathway for patients who have experienced delirium following hospitalisation [6, 7] unlike those available for cardiac [8] or stroke patients [9].

Delirium is among Australia’s most common hospital-acquired complications (35.7 per 10 000 admissions), costing an estimated A$8.8 billion annually [10–12]. The Australian Delirium Clinical Care Standard (Quality Statement Eight) recommends individualised discharge care plans and timely General Practitioner or specialist follow [12, 13]. This aligns with the UK ‘care closer to home’ agenda, which similarly emphasises community-based care [14, 15], yet in practice, implementation remains limited [12, 16].

Rehabilitation programmes are essential in health care because they help people regain independence and quality of life while reducing complications, readmissions and long-term health-system costs. For example, cardiac rehabilitation programmes have been in operation for over 50 years [17], and evidence demonstrates that they reduce mortality by 47%, myocardial infarction risk by 31% [18] and death or hospital readmission by 21% [19], along with improvements in health-related quality of life (standard mean differences = 0.13–0.47) [20]. Similar benefits have been observed for stroke [21–23], dementia [24] and traumatic brain injury [25] rehabilitation programmes. With an ageing population and increasing recognition of delirium and its poor long-term outcomes, rehabilitation strategies for patients who previously experienced delirium could provide substantial health and cost-saving benefits [7, 26].

To our knowledge, there are currently no evidence-based rehabilitation programmes for delirium embedded in health care systems. Given this gap, we sought to use coproduction methodology to develop the Help After Leaving hospiTal (HALT) delirium rehabilitation programme. Qualitative analysis of data from focus groups and interviews with people who experienced in-hospital delirium (*n* = 3), their support persons (*n* = 6) and health workers experienced with delirium patients (*n* = 28) were used to inform initial programme design [27, 28]. The results suggested to use a multi-disciplinary approach, address delirium knowledge gaps through education, engage support persons, tailor the programme based on needs and preferences, acknowledge the challenges of returning to baseline levels of function and promote autonomy and self-management. These findings were similar to those of the Recovery after an Episode of Delirium (RecoverED) research group, which developed a delirium rehabilitation programme in the UK through collaborative involvement of patients and the public, [7, 26, 29], although this work is no longer active. Analysing interview data with a similar group of stakeholders (*n* = 46), they also highlighted the need for education, involving support persons, promoting personhood and tailoring and addressing multiple lifestyle factors affecting delirium recovery [6], with a feasibility trial showing promising outcomes despite challenges in recruitment and retention [30, 31]. Further, feedback from other rehabilitation programmes such as in cardiac [32–34] and stroke [35, 36] settings, and older adults’ reflections on co-designed well-being programme [37] suggest a multidisciplinary, tailored and flexible approach, and emphasise the value of a modular programme, ongoing support, strong patient–facilitator collaboration, support persons’ education, psychosocial support, physical activity, nutrition and medication review components.

HALT has been developed to be multicomponent, with three essential modules, (delirium education, vision and hearing and medications) and five optional modules (cognitive engagement, sleep, nutrition, physical functioning and wellbeing). Modules are delivered sequentially and run for between two and eight sessions. The selection, order and duration of each module are tailored to individual needs through appropriate screening tools. The programme is facilitated remotely by trained health facilitator while participants remain at home, and it can be completed with or without a support person. The programme will commence 2–4 weeks after discharge.

After designing this programme structure and modular content, we next sought feedback in a series of workshops to review the draft programme. A similar coproduction step was completed by the RecoverED team; however, their feedback results are not yet published [6, 29–31]. Therefore, this paper will provide insights from this important coproduction step, to apply to the HALT programme and also to other delirium rehabilitation programmes around the world. The aim of this study is to report feedback on structure, content, resources and engagement strategies, from health professionals and consumers of the draft HALT rehabilitation programme for delirium. Our key research questions included:

1. How can the HALT programme be enhanced to better support and engage programme users?
2. What are the broad challenges and enablers that could affect implementation of the HALT programme?
3. Is the programme structure, including modular delivery and resources, accessible to older people who have experienced delirium in hospital?

## Methods

### Participants

Twelve health professionals and four consumers participated in the workshops. Consumers were defined as individuals with lived experience of delirium, either as patients or support persons. The patient group included individuals aged 60 years and older who had experienced delirium and had no clinical diagnosis of dementia. Support persons were defined as an adult (aged ≥18 years) friend, volunteer carer, relative or family member who spent at least 1 h per week with a person who had experienced delirium, for at least the first 6 months after the delirium occurred. The health professionals group was composed of multidisciplinary professionals with experience working with patients with delirium. Detailed recruitment procedures and eligibility criteria are reported elsewhere [27, 28]. All participants who had taken part in the programme development focus groups and interviews were invited to attend [27, 28]. Two health professionals with no prior involvement in the earlier programme development also participated, as they heard about it through colleagues and wanted to attend. See Table 1.

**Table 1:** Demographic information of the participants.

Characteristic	Health professionals (*n* = 12)	Support persons (*n* = 3)	Person who experienced delirium (*n* = 1)
Sex			
Female; *n* (%)	9 (75)	3 (100)	0 (0)
Age (years); *n* (%)			
20–29	4 (33)		
30–39	4 (33)		
40–49	4 (33)	1 (33)	
50–59			
60–69		1 (33)	
70–79		1 (33)	
80+			1 (100)
Profession; *n* (%)			
Geriatrician	1 (8)		
Nurse	1 (8)		
Physiotherapist	5 (42)		
Exercise physiologist	2 (17)		
Occupational therapist	3 (25)		
Experience working with delirium (years)			
(mean ± SD)	1.9 ± 1.0		
Ethnicity; *n* (%)			
Caucasian		3 (100)	1 (100)
Education; *n* (%)			
High school	0 (0%)	1 (33)	0 (0%)
Tertiary education	12 (100%)	2 (67)	0 (0%)

### Design

A descriptive qualitative study was conducted. Each participant was involved in one workshop. We employed a co-production approach, which actively engages stakeholders throughout the research process [38].

### Materials

A semi-structured workshop guide (Supplementary Appendix 1) was reviewed in advance by the HALT research team (which included a Consumer Representative). The guide consisted of six questions for health professionals and seven for consumers, focusing on their reflections on the design and delivery of the programme, the module contents and formatting and how the programme can best support the study participants.

### Procedure

This study had ethics approval from the Central Adelaide Local Health Network Research Ethics (Ref 2024/HRE00115) and the University of South Australia Human Research Ethics Committee (Ref 206536). Written informed consent, including audio recording was obtained from all participants prior to commencement of the workshops. Two audio recorders were used to record the workshop discussions.

Five in-person workshops were conducted between August and September 2025. Three workshops were held with health professionals at two hospitals (Modbury and Lyell McEwin) in a large South Australian metropolitan health network, and two workshops were held with consumers at the Adelaide University Magill campus (see Supplementary Appendix 1 for further details).

Group ground rules were proposed by facilitators and reviewed by participants. Facilitators used active and reflective listening techniques to encourage engagement from all participants. Each workshop began with a 15-min PowerPoint presentation outlining the HALT programme, followed by an opportunity for participants to seek clarification to ensure a shared understanding. A brief brainstorming vignette (see Supplementary Appendix 1 for further details) was used to prompt discussion, and observational notes were taken to provide contextual information alongside verbal contributions.

Once a shared understanding of the HALT programme had been established, the first question was introduced and open discussion followed. Probing questions were used to clarify responses and elicit further detail. Although each question was allocated 5–7 min, timing remained flexible to accommodate the natural flow of discussion. Key points were summarised regularly to confirm accurate interpretation and extend the discussion where appropriate. Copies of the intervention module resources were provided to prompt discussion on content and formatting throughout the workshop.

Workshops were facilitated by Y.T. (PhD candidate) as the lead facilitator, supported by C.R.D. (Programme Manager, PhD) and M.J.H. (qualitative research expert, PhD). Sessions were conducted in English, in quiet rooms, and held separately for health professionals and consumers to minimise power imbalances during discussions [39]. The health professional workshops lasted approximately 1 h and 30 min, whereas the consumer workshops averaged 2 h and 30 min. Refreshments were provided, and consumer participants received a $50 AUD gift card at the end of the session.

### Analysis

Two facilitators (Y.T. and C.R.D) transcribed the recordings verbatim using the Microsoft Word Transcribe function. Participants’ identifying information was removed and transcripts were reread for accuracy and data familiarisation. Field notes were used to provide contextual details not captured directly in transcripts. Inductive coding was then undertaken using NVivo qualitative analysis software (Version 14) [40]. The data were double-coded (Y.T.), and verification checks were completed by another facilitator (C.R.D.) against the original recordings. Data saturation was considered achieved after preliminary codes were reviewed with the wider team. A thematic analysis approach was used to generate the findings [41, 42]. The coding process was guided by the workshop aims and the overarching research question. Facilitators engaged in iterative discussions to reach agreement on the coding, after which the research team reviewed the codebook. Similar categories were subsequently organised and clustered using an Excel spreadsheet. Once consensus was reached, themes were developed and refined through consultation with the broader research team. The health professional and consumer data were coded separately; however, the final themes are presented together, as no meaningful differences were found that warranted separate reporting.

Verbatim quotations were selected to illustrate and support each final theme. The study adhered to the Consolidated Criteria for Reporting Qualitative Research [43].

## Results

Five themes were generated from thesedata.

### Theme 1: Personalisation and provision of enhanced support

Participants perceived the programme’s flexible and tailored structure as highly relevant and emphasised the importance of accommodating individual differences in needs, preferences and capacities. Recognising individual variability and incorporating personalisation were viewed as key factors for enhancing motivation and engagement, particularly within the physical function module, where substantial variability in functional capacities was noted.

‘Everybody needs choice of what they do, and with choice will further enhance engagement and participation’. Support person #1.

Health professional participants particularly identified phone-based support as a suitable delivery method for individuals with limited mobility or social isolation. They highlighted that these individuals are commonly missed in centre-based rehabilitation programmes due to mobility constraints and limited support systems. Remote support was therefore perceived to address some of these challenges. Participants also considered the programme content, including regular one-on-one phone or virtual contact and motivational interviewing, to provide valuable emotional support and help address the loneliness experienced by participants (see Supplementary Appendix 1 for further details).

‘Yeah, because I guess the people that you would get included into this programme may be the ones that are socially isolated, that don’t leave the house very often. There’s a lot of them, can be quite lonely in that degree, and you do pick up a–a good chunk of people that would get missed otherwise in the community to. Hmm, so I guess even just the phone call to include those type of patients, I think that’s a success’. Exercise physiologist #1.

While participants agreed that remote-based support was appropriate, they also emphasised the importance of offering alternative delivery modes, including face-to-face, audio-visual, one-on-one and group-based options, to accommodate diverse learning styles, impairments and personal preferences.

‘So generally, I think phone call was just gonna [sic] have to be, and that’s fine. I think if there’s other ways to tap into a face to face or that tele [health], then great’. Occupational therapist #1.

Participants found that the programme’s approach of incorporating needs-based recommendations (i.e. poor nutrition flags the nutrition and hydration module), while also enabling participants to choose which modules to complete and the partial sequencing of these modules, was an important strategy for supporting engagement, particularly among older adults who may experience cognitive, literacy or socio-economic related challenges. To further support engagement, participants suggested more frequent phone contact and page-by-page guidance, especially for essential modules (see Supplementary Appendix 1 for further details).

*‘*Hmm, all regular sort of patients that we get through, you’re more socially vulnerable, you know, your health literacy, socioeconomic levels are really quite on the lower side. So, yeah, that first sort of holding their hand through the first part may be beneficial, to get that buy-in, to get them sort of on track, and then winging it’. Physiotherapist #1.

### Theme 2: Beyond involvement, supporting support persons is essential

Workshop participants agreed with the programme’s planned inclusion of support persons, when available, provided patient consent is obtained. Support person involvement was identified as a particularly important strategy given the programme’s remote support and self-directed nature. Participants emphasised that support person engagement is associated with improved participant motivation, adherence and overall outcomes (see Supplementary Appendix 1 for further details).

‘Their support people to be like, “Alright, let’s sit down, let’s go through this today,” will motivate them’. Physiotherapist #2.

Both health professional and consumer workshop participants agreed that an educational and wellbeing resource for support persons would be useful, including education about delirium, practical guidance on offerng support, strategies to manage caregiving-related stress and information about available resources to assist with both caregiving tasks and emotional wellbeing.

‘I think a carers module on delirium and what it is and what it looks like and, you know, journey will look like to getting back and what’s normal and what’s not normal because I think a lot of carers aren’t familiar with the presentation and what it, what it does look like’. Exercise physiologist #1.

One area of tension was the desire to include support persons while maintaining a focus on the person with delirium. Participants were cautious against a parallel, support person-specific module, given the programme’s primary focus on the delirium patient.

‘I think you should target the patients. I think the carer is coming along as support and helping the patient to go through the journey, for them to understand’. Physiotherapist #2.

Workshop participants highlighted the need for additional support mechanisms for those in the programme who do not have a support person.

‘Ideally, there would be a carer with them to do the programme, bit more of a challenging [sic] when they don’t have a support person’. Geriatrician.

In addition, some suggested that optional, separate meetings between support persons and programme facilitators could provide an opportunity to discuss participant-specific conditions and concerns. Across discussions, participants emphasised that support persons are frequently overlooked within healthcare processes, underscoring the importance of their active inclusion and their potential to assist with the programme.

‘We do get a lot of family members, whoever’s providing that support or care, voicing carer stress. So, I think it is something that they would be seeking to a certain degree, like if they are worried about their loved ones, particularly an acute delirium can be really distressing for the family members and the carers to witness’. Occupational therapists #1.

### Theme 3: Be a trusted partner

This theme captured the importance of establishing trusting partnerships between the HALT programme and the hospital, as well as between programme facilitators and programme users.

Health professionals emphasised the need for strong links between acute-care hospital settings and the rehabilitation programme, to ensure that patients and support persons are informed about the HALT programme and receive referrals at discharge. Familiarity with the programme would enable hospital staff to advocate for its inclusion during discharge planning (see Supplementary Appendix 1 for further details).

‘I think that screening and referrals from hospital will be really important, people to be aware of the programme for the entire. So, you know, planning appropriate referrals. so having it be visual in the hospital’. Geriatrician.

‘I think when you roll it out, you’re gonna [sic] have to spread it everywhere. Talk to every ward, because there’s gonna [sic] be delirium in every ward. Hmm, involve them, the managers. Hmm, put it on the weekly meeting schedules that they all have’. Nurse.

Embedding referral prompts and recruitment flags within electronic medical records and discharge documentation was identified as important, if possible.

‘Is there any way to flag in the EMR [Electronic Medical Records]? that just to automatically have a text message or a letter to the patient saying, “you have had delirium, there is this education programme for you to participate in”’. Nurse.

Establishing rapport with programme users was considered critical for maintaining motivation and ongoing engagement, with consistent communication, respect, attentiveness and programme facilitator skills identified as key contributing factors.

‘I think that’s the biggest way that you create people engaged, is that phone call, that weekly check-in, when you slowly build the rapport with them’. Nurse.

### Theme 4: Overcoming barriers to optimise engagement

Workshop participants identified several barriers to older adults’ involvement and engagement in the programme, including low motivation for behaviour change, entrenched habits and the self-directed and remotely delivered nature of the intervention. Factors such as cognitive difficulties, literacy status, language barriers and the availability of a support person were also predicted to influence participants’ readiness and ability to engage (see Supplementary Appendix 1 for further details).

‘I think motivation will be your biggest challenge. Particularly for people that live out this way who aren’t, yeah, the most proactive in a lot of ways, and have, like, entrenched habits that aren’t helpful for their health’. Occupational therapist #1.

Participants reported that printed materials were less engaging than in-person support. Using interactive activities and going through each booklet page-by-page were suggested as ways to better engage with written materials. They also expressed concerns that completing the three essential modules alongside additional optional modules could be time-intensive, which may increase the risk of participant disengagement.

*‘*Because if you’re giving these and it’s not overly interactive because it’s just printed–well, then they can read it, but what do I do with this information? Where is it? If it’s interactive on the internet–or I know you need to cater for a broad population, which is also another challenge within itself–and the interactive thing on the computer may be a little bit more involved, with games or different things along the way’. Support person #1.

Workshop participants agreed on broad programme strategies to enhance engagement, including individual goal setting, affirming achievements and raising awareness of delirium.

‘I think just goal setting would be probably the biggest one. Just, hmmm, getting them to create their own goals around each topic’. Exercise physiologist #1.

‘They don’t wanna [sic] get it again. Dad didn’t want to get it again, so raising awareness will be a motivating thing’. Support person #3.

Participants added that clearly communicating the programme’s benefits, and the possible harms of delirium after hospital would enhance motivation.

‘Present it to them as something that is going to be worth-while. “Did you like having delirium?” “No.” “Well, do you want delirium again?” “No.” Well, pretty self-explanatory to me, I think it has to be really, really, really explained to them’. Support person #2.

Other suggestions to enhance engagement included involving the general practitioner, and longer and more frequent contact points.

‘I would honestly say the more the better. So, if, yeah, if it can be afforded to be more, like two to three times a week’. Occupational therapist #2.

### Theme 5: Comprehensive content and easy to read materials

Participants described the programme modules as comprehensive, well structured and closely aligned with the needs of people who have had delirium, noting that the content reflected issues raised during the focus group discussions (see Supplementary Appendix 1 for further details).

*‘*I think they’re good, I can’t think of anything else that you could possibly cover’. Support person #3.

The modules were considered interesting and useful, with many participants indicating they would recommend the programme to others.

‘This would have really appealed to Dad, how you have put this together’. Support person #2.

The booklets were viewed as easy to read and visually appropriate for older adults, with well-designed spacing, colours, font size and images. Participants preferred the A4 format over A5. Although the paper-based format was generally well received, participants recommended distributing modules gradually rather than as a single bound booklet to avoid overwhelming users.

‘Font size is great. I love the colour; I love the pictures. It’s not too wordy’. Nurse.

They valued the inclusion of lived experience stories, which were perceived to instil hope and enhance engagement, and recommended incorporating additional narrative examples illustrating delirium rehabilitation strategies throughout the modules.

‘I like the story pretty much, because a bit further on in pages, he gets better’. Person who experienced delirium.

Consumer workshop participants highlighted confusion they experienced between delirium and dementia and suggested the differences should be clarified earlier in the education module.

*‘*Actually, putting that page somewhere towards the front really would be good. I think that, that’s really, really important page because when you tell someone they’ve got del- delirium, they immediately think they have dementia’. Support person #2.

Health professional workshop participants stressed the importance of enhancing physical functioning capacity and suggested reframing the physical activity module to better reflect this focus. Both consumers and health workers also suggested some (e.g. cognitive engagement) or even all the non- essential modules may need to be made essential. Overall, both consumers and health professionals responded favourably to the programme.

‘I think it’s going to be pretty successful just judging what you have. I think it’s so unique’. Exercise physiologist #1.

## Discussion

Feedback on the HALT delirium rehabilitation programme identified that participants agreed with the module topics, resource design, remote facilitation approach and involvement of support persons. Ideas to enhance engagement and implementation included strengthening relationships with hospitals, enhancing assistance for support persons through dedicated resources and refining elements of the resource booklet.

As demonstrated in Themes 1 and 4, key feedback suggested flexibility in timing and duration of the programme, as well as which modules are compulsory, could better support users’ individual needs and preferences. As a result, completing certain HALT programme modules will no longer be mandated; rather some modules are considered more essential, but not compulsory. Further, the programme will embed timing flexibility in terms of module duration (orienting around session number rather than week number) and permit varied-length breaks between modules (Table 2).

**Table 2:** Changes made following workshop feedback.

Themes	Changes made following feedback
Personalisation and provision of enhanced support	Breaks between modules
	Flexible timing and duration of modules Range of delivery options (phone call or virtual supplemented by text or email)
	Frequent phone contact
	Page-by-page guidance
	Additional lived experience stories
	Essential (rather than compulsory) modules
Beyond involvement, supporting support persons is essential	Family and Friends booklet
Be a trusted partner	Strengthening relationships with hospitals and health professionals
	Strengthening rapport with consumers
Overcoming barriers to optimise engagement	Page-by-page guidance
	Breaks between modules
	Communication of programme benefits and potential harms of delirium
	Focus on physical functioning capacity
	Frequent phone contact
Comprehensive content and easy to read materials	A4 format
	Early clarification of differences between delirium and dementia
	Gradual distribution of modules rather than a single bound package

These changes build support via personalisation (individual tailoring), reflecting Theme 1. The Whole Health STEPS peer-delivered wellness programme for US veterans (*n* = 16) study [44], along with HALT focus groups and interviews [27], emphasised flexible scheduling to meet users’ needs, optional shorter session durations, goal setting, the use of programme booklets and building and maintaining rapport to support via personalisation. Evidence from RecoverED feasibility trial [31], along with cardiac [33] and stroke [45] rehabilitation programmes also demonstrate that adequate support for programme users, including individualised goal setting, improves engagement and adherence and facilitates lifestyle behaviour change. Changes made to the programme in response to this feedback, such as incorporating additional strategic storytelling to enhance relatability, may further support user engagement.

The need to assist support persons was also highlighted in Theme 2. Research has demonstrated that, while support persons are often willing to help [31], they frequently experience significant gaps in delirium knowledge and high levels of caregiving distress [6, 46, 47], findings that were also reflected in our qualitative study [27], underscoring the importance of targeted support. Previously, information for support persons was embedded within patient resource booklets. Based on feedback, this content has been moved into a separate Family and Friends booklet, providing more tailored and targeted written resources for both support persons and patients.

Another opportunity for programme improvement identified through Themes 1 and 4 were ways to specifically enhance user engagement, by acknowledging and providing information and strategies to directly address barriers. Participants highlighted that the remote mode of delivery, along with cognitive challenges following delirium, limited support networks, existing health conditions and the motivation required to adopt lifestyle changes in later life, may hinder engagement. Similar challenges have been reported in cardiac [8, 48], stroke [45, 49], mild cognitive impairment [50] and exercise [51] rehabilitation programmes. Feedback from Whole Health STEPS also raised similar barriers to engagement, including low motivation for making health and lifestyle changes, lack of access to a working phone and competing immediate needs (e.g. homelessness, basic healthcare), which could lead to disengagement.

Remote delivery may, however, help overcome accessibility and cost barriers commonly associated with centre-based rehabilitation programmes [50]. The RecoverED feasibility trial demonstrated that providing support to participants in their own homes was feasible, while also highlighting that in-person support posed challenges for inclusive delivery, particularly for those in remote and rural areas, and suggested alternative strategies to address this [30]. Remote support could offer this opportunity, which also aligns with the UK agenda of ensuring care closer to home [14]. This is particularly important in the context of delirium in older adults; accessibility of intervention programmes has been identified as a key factor motivating older adults to engage in lifestyle interventions [50]. Previous research has shown that older adults report both telephone-based [52] and home-based [53, 54] support as helpful.

Remotely facilitated programmes such as Maintain Your Brain (*n* = 6104) and APPLE-Tree (*n* = 746) have utilised digital support to achieve succesful lifestyle behaviour change in older people. Maintain Your Brain prevented cognitive decline in at-risk older adults over 3 years (between group difference Z score 0.18) [55]. Although APPLE-Tree trial did not reach statistical significance, it suggested a small improvement in cognitive decline outcomes in the intervention group after 12 months [56]. In contrast, a web-based combined cognitive and physical training intervention in patients who had experienced delirium or subsyndromal delirium during intensive care unit admission (*n* = 153) showed no improvements in cognitive performance, quality of life or symptoms of depression and anxiety compared with the control group at 3 and 6 months [57].

Retaining the remote delivery approach, refinements to the HALT programme to address barriers and better support users include offering more frequent or longer contact points. The physical activity module is reoriented toward improving functional outcomes, with greater emphasis on balance and strength, while the distinction between delirium and dementia is introduced earlier in the education module to reduce confusion. In addition, facilitators will receive training in motivational interviewing to better support behaviour change among programme users. This could help improve the low motivation and engagement in physical and cognitive intervention observed in RecoverED feasibility trial [31].

Building good relationships is linked to supporting engagement as well as implementation and was reflected in Theme 3. Strong linkages with hospitals, may enhance programme user referrals and help address recruitment challenges noted in the RecoverED feasibility trial [58]. Health systems, networks and health professionals that are aware of the programme could facilitate streamlined referrals at discharge, potentially reducing missed opportunities [59]. This is particularly important as lack of knowledge about available programmes is one of the most common barriers to participation in cardiac [60] and stroke [61] rehabilitation programmes. Consistent with this study findings, the RecoverED feasibility trial also found that rapport and trust between participants and the intervention provider were highly valued, encouraging and motivating users and showing promise for integrating the intervention into daily life [31]. Furthermore, one-to-one programme support could provide emotional support for participants experiencing loneliness [62].

### Implications, strengths and limitations

In Australia, when a patient is discharged from hospital, a discharge summary outlining the diagnosis, treatments, medication changes, follow-up and ongoing care recommendations should be shared with the patient’s General Practitioner. Depending on the patient’s needs, the hospital may also arrange General Practitioner or specialist follow-up and referrals to community nursing or allied health services to support a safe transition home. However, these are not implemented consistently in practice [16, 63]. Current post-discharge support is provided through a combination of Federal, State and Local Health Network funded programs, including Transition Care Program, Hospital in the Home, the Commonwealth Home Support Programme, Department of Veterans Affairs Community Nursing and geriatric outreach services, however, these focus primarily on short-term recovery and require referral and eligibility assessment [16]. Primary care is largely General Practioner-led, and delirium is not consistently noted on discharge summaries provided to General Practitioner. Delirium-specific post-discharge services remain scarce, although emerging models such as specialist delirium clinics in Queensland, demonstrate growing recognition of this unmet need. Structured delirium-specific follow-up and long-term rehabilitation post-discharge remain a significant gap in Australian healthcare system. We hope this work will stimulate further research in delirium rehabilitation and support implementation of the Australian Delirium Clinical Care Standard (Quality Statement Eight), improving post-discharge care for those who have experienced delirium.

This feedback is part of the co-production process of the HALT programme, but the lessons can be applied more broadly to other delirium rehabilitation programmes around the world. A key strength of this study was the inclusion of consumers and health professionals in the programme’s co-production process. While stakeholder involvement in intervention design is increasingly recognised as best practice [29, 39], how feedback informs programme refinement is often poorly documented. In this study, reflections from both consumers and health professionals were embedded as a core component of the co-production process, providing valuable opportunities to optimise the intervention and enhance the programme’s practicality. However, several limitations should be acknowledged. Recruitment of participants with lived experience of delirium proved challenging, resulting in limited representation of this group and greater influence of health professional and support person perspectives on the findings. A similar challenge was reported in the RecoverED feasibility trial, affecting the generalisability of findings [30], and may be attributed to the frailty and physical and cognitive difficulties commonly experienced following in-hospital delirium [2, 64]. The workshops were conducted as a single, non-iterative process, which limited opportunities to refine programme content through successive rounds of participant feedback. Time constraints and participant availability restricted the depth of discussion, and we were unable to go through each booklet using a page-by-page approach.

The vast majority of workshop participants were involved in earlier programme development [27]. While this helped to verify that the programme reflected their views, perspectives and needs, it may have influenced their evaluation and limited new perspectives. The health professional sample consisted primarily of early-career professionals working in delirium care; more experienced professionals may have offered different perspectives. Although all participants had experience working with delirium patients, most workshop participants were from physiotherapy and exercise physiology backgrounds, which may have influenced the depth of the data, as nurses and doctors typically play a central role in deliriumcare.

**Figure 1:**
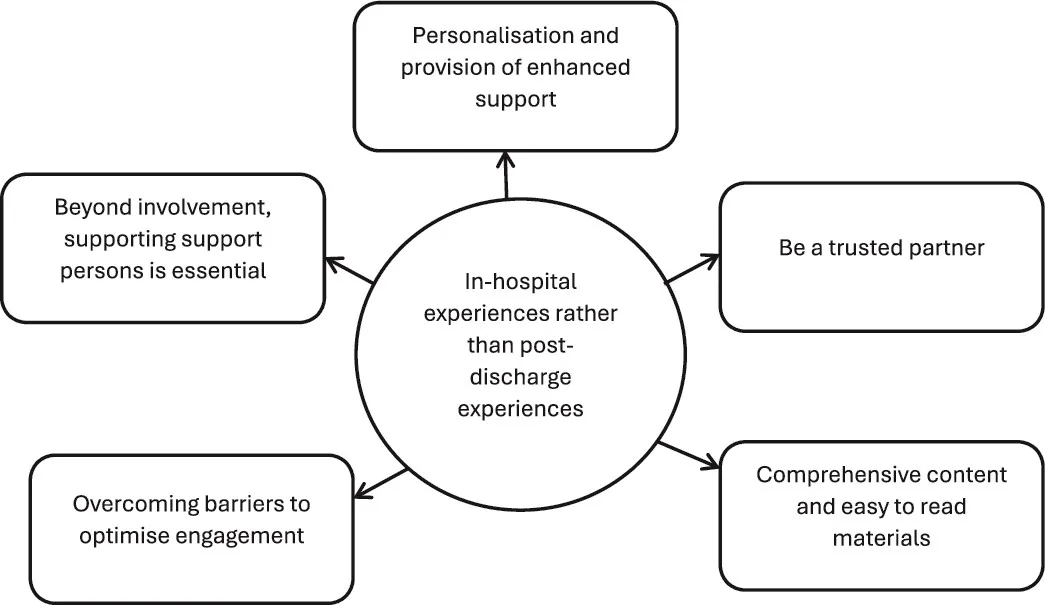
Themes underpinned by in-hospital experiences rather than post-discharge experiences.

Similar to our focus groups [27], consumer workshop participants’ reflections were primarily shaped by their in-hospital experiences rather than post-discharge experiences, underpinning all themes (Figure 1). This may be due to the brain’s tendency to retain strongly emotional experiences [65]. Finally, despite efforts to recruit participants from diverse backgrounds, the consumer sample lacked cultural diversity, and data on the ethnicity of health professionals were not collected. Findings should be interpreted in this context and may not apply to different cultural groups or health systems in other countries.

This study report consumer and health professional feedback on a co-produced delirium rehabilitation programme for older adults. Incorporating this feedback enabled refinements that may strengthen the feasibility of the HALT programme during subsequent piloting. This is particularly important given evidence from cardiac rehabilitation showing that many eligible individuals do not participate despite demonstrated benefits, and that non-adherence rates remain high [66–68]. Actively integrating feedback from programme users and service providers can help identify potential implementation challenges early and improve the likelihood of success during feasibility testing [44]. Further feedback will be sought in a pilot-test of the HALT programme currently underway, and with future funding, we hope to develop web resources to support remote access.

## Conclusion

This study demonstrates that personalisation, assisting support persons, strong hospital partnerships and addressing barriers are essential for engaging older adults who have experienced delirium in rehabilitation programmes. Programme format and content were broadly endorsed. Iterative changes made overtime to the HALT programme in response to feedback are likely to improve its relevance and usability. Taken together, our aim is for HALT to assist people who experienced delirium via structured phone calls or virtual, interactive activities, goal setting, the integration of lay case studies and the development of rapport through motivational interviewing, to ultimately promote better health after delirium.

## Supplementary Material

aa-26-1438-File002_afag269
