# Supplementary material for: Consumer and health professional reflections and feedback on the Help After Leaving hospiTal delirium rehabilitation programme
Source: Age Ageing. 2026 Sep 16;55(9):afag269. doi: 10.1093/ageing/afag269 (PMC13580086; doi:10.1093/ageing/afag269)
Supplement: Supplementary file 1 [file aa-26-1438-file002_afag269.docx]

**Appendix 1**

**Consumer and health professional reflections and feedback on the Help After Leaving hospiTal delirium rehabilitation programme**

**Table of Contents**

Contents

[Workshop guide questions	3](#_Toc236300008)

[Health professionals	3](#_Toc236300009)

[Consumers	3](#_Toc236300010)

[Brief brainstorming vignette	4](#_Toc236300011)

[Workshop attendance	4](#_Toc236300012)

[Phone or virtual contact as emotional support and helping to address loneliness.	4](#_Toc236300013)

[Table 2. Summary of codes, sub-themes, themes and demonstrative quotes	6](#_Toc236300014)

# Workshop guide questions

## Health professionals

Q1. What are your first impressions of the eight modules?

**Prompts**: Do you think we’ve covered everything? Are we missing anything? Is anything unnecessary?

Q2. How can we best support patients in this remotely supervised delirium rehab programme?

**Prompts**: How should we interact with carers? How do we ensure they are well enough to participate? Remind them that the rehab programme are the 8 modules.

Q3. Patients will be recommended some modules if they are in need and willing to change a behaviour. For example, if someone is malnourished and willing to change their diet, they will be recommended the nutrition and hydration module. What do you think of this approach?

**Prompts**: If someone not in need, should they be given the opportunity if they want? Should carers be welcomed to undertake the activities?

Q4. How can we make this programme a success? What factors are most important?

**Prompts**: What are our biggest challenges? How can we overcome these challenges? Thoughts about design and delivery?

Q5. Looking at these resources, what are your thoughts on the format, colours, activity types etc?

**Prompts**: Are there any changes you would suggest? Are they simple and easy to understand?

Q6. Anything you would like to say or add?

## Consumers

Q1. What are your first impressions of the eight modules?

**Prompts**: Do you think we’ve covered everything? Are we missing anything? Is anything unnecessary?

Q2. How can we best support people in this remotely supervised delirium rehab programme?

**Prompts**: How should we interact with carers? How do we ensure they are well enough to participate? Remind them that the rehab programme are the 8 modules.

Q3. People who are referred to the programme will be recommended some modules if they are in need and willing to change a behaviour. For example, if someone is malnourished and willing to change their diet, they will be recommended the nutrition and hydration module. What do you think of this approach?

**Prompts**: If someone not in need, should they be given the opportunity if they want?

Q4. Would you do this programme or recommend it to others? If yes, why? If not, why?

**Prompts**: Thoughts about design and delivery? What would we need to make you want to do it?

Q5. How can we best involve carers?

**Prompts**: Should there be separate resources? Should carers be offered to do the same programme using the same resources?

Q6. What do you think about these resources?

**Prompts**: What are thoughts about the colour, format, activity types, simple and easy to understand, etc? What do you feel about the term “goal setting” when changing behaviours?

Q7. Anything you would like to say or add?

# Brief brainstorming vignette

Mr. Smith had a delirium in hospital and was screened for which modules he needed to complete. We recommended that he complete the nutrition and hydration, and sleep modules, and he was happy to do so. Mr. Smith then started the programme 4 weeks after discharge from hospital, beginning with the following modules sequentially: Delirium Education, Medications, and Vision and Hearing. We provided him with support via phone call. After this, he started the sleep module (6 weeks), then the nutrition module (8 weeks). The programme took him 18 weeks in total. What do you think of this approach?

# Workshop attendance

Five in-person workshops were conducted between August and September 2025. Three workshops were held with health professionals at two hospitals (Modbury and Lyell McEwin) in a large South Australian metropolitan health network; with five, three, and four participants attending the first, second, and third workshops, respectively. Two workshops were held with consumers at the Adelaide University Magill campus, with two attending each workshop. Notably, 33% of consumers returned to participate in a workshop from the focus groups/interview stage, and 43% of health professionals [27].

# Phone or virtual contact as emotional support and helping to address loneliness.

Table 1: Conversation between a person who experienced delirium and the workshop facilitator illustrating the importance of programme phone calls in addressing loneliness.

Person who experienced delirium: “And yeah, it maybe leads on to, ‘Should there be more talking amongst friends and things like that?’ because we are becoming more and more isolated.”

Facilitator: Absolutely.

Person who experienced delirium: “I go back to when I was a child—it was a wonderful life, you know. Everybody in the town knew each other. It didn’t matter if they were older people or younger; they’d still say hello to you, yeah.

Facilitator: “Yeah.” [expressing understanding]

Person who experienced delirium: “Quite interesting when I look back at that, at my own life. And so, yeah, people used to talk all the time.

Person who experienced delirium: “Have you got a roster of dates or anything [for when to start the programme]?”

Facilitator: “So, at this stage, the programme isn't live. I would say it's not live. We're still working on it. Hmm, the programme probably won't start properly until probably even this time next year.”

Person who experienced delirium: “So, we won't have phone calls for a while yet.”

Facilitator 2: “Not for a while.”

Person who experienced delirium: “That's all right.” [looks sad] “I'll probably forget about it by then.”

# Table 2. Summary of codes, sub-themes, themes and demonstrative quotes

| **Themes** | **Codes/ sub-themes** | **Demonstrative quotes** |
| --- | --- | --- |
| Personalisation and provision of enhanced support | Choice on the module | *“Everybody needs choice of what they do, and with choice will further enhance engagement and participation.”* *Support person #1* |
|  | Personal conditions | *“I guess it really depends on how long you spent in hospital too, and the decline, and being weak and isolated apathetic.”* *Support person #1* |
|  | Self-paced | *“Bit making it to be like self-paced, so they can do it as quick or as low as they want, I think it’s gonna be the best way.”* *Exercise physiologist #2* |
|  | Capacity | *“In saying that, with the physical function module, you'll have some people that are independent, unaided and go walk 500 metres, then you probably have some others that can only walk to the bathroom, and so, yeah, how to identify, how you gonna identify those who and Who's appropriate for it and stuff. I think the other ones like cognition, sleep and that sort of stuff, anyone probably be able to do those. Humm, it's just with the function of physical limitations” Exercise physiologist #1* |
|  | Support | *“Maybe once a week for the proactive people and more frequently for those who are lagging behind.”* *Nurse* |
|  | Choice on the module | *“Hmm, and I guess it’s really in consultation with whoever’s doing that, I suppose to work out, “Well, I want you to do this one because of your situation, then you can do these two or three others.” And then it’s really up to that person to lead – which, in what order specifically.”* *Support person #1* |
|  | Choice of mode of delivery | *“So generally, I think phone call was just gonna have to be, and that's fine. I think if there's other ways to tap into a face to face or that tele, then great.”* *Occupational therapist #3* |
|  | Missed group | *“Yeah, because I guess the people that you would get included into this programme may be the ones that are socially isolated, that don't leave the house very often. There's a lot of them, can be quite lonely in that degree, and you, you do pick up a–a good chunk of people that would get missed otherwise in the community to. Hmm, so I guess even just the phone call to include those types of patients, I think that's a success.”* *Exercise physiologist #1* |
|  | Holding hand | *Hmm, all regular sort of clientele patients that we get through, you’re more socially vulnerable, you know, your health literacy, socioeconomic levels are really quite on the lower side. So, yeah, that first sort of holding their hand through the first part may be beneficial, to get that buy-in, to get them sort of on track, and then wining it.”* *Physiotherapist #1* |
|  | More is the better | *“I would honestly say the more the better. So, if, yeah, if it can be afforded to be more, like two to three times a week, would be–and I guess you gauge that with the patients themselves as well. They might, yeah, might not want that many, or they might.”* *Occupational therapist #2* |
|  | Different mode of delivery | *“You might have to have a different mode of delivery apart from, you know, visual, hear, read. You might have to have a different mode depending on they can be better on. I don't know what are their mode- every older people are different, hmmm, and I think then you need to identify what mode is better for them. It could be a one-on-one, it could be hearing. Forget about the book, because they're never going to read it, or like someone just have to show them what to do. I don't know, I don't know” Physiotherapist #1* |
|  | Options | *“Would there be options for both of them as in, you know, whether they're actual kind of the goal-setting element is [inaudible] into on a one-on-one kind of thing, but some of the other parts of different sessions are offered group sessions?”* *Physiotherapist #1* |
| Beyond involvement, supporting support persons is essential | Carer involvement | *“Get their carer or home support person involved if they've got home support access.”* *Support person #1* |
|  | Engaging carers | *“I think engaging their support person is gonna be really important.”* *Physiotherapist #3* |
|  | Carers are valuable | *“Probably something that a carer would be most valuable for, someone to prompt them.”* *Nurse* |
|  | Carer involvement | *“Probably carers be part of the whole process right from the consent and the screening, on the ward, potentially.”* *Occupational therapist #3* |
|  | Motivated carer | *“So maybe one of the ways we can motivate the patient is by relying on the motivated carer in a way.”* *Exercise physiologist #2* |
|  | Carer motivates | *“Their support people to be like, “Alright, let’s sit down, let’s go through this today,” will motivate them.”* *Physiotherapist #2* |
|  | Carers support outcomes | *“You know what, when you involved them, you would see better outcome.”* *Exercise physiologist #1* |
|  | Targeting carer | *“Even if you don't have the person that's got the delirium, at least you've targeted the family and at least you've educated the family into what it is.”* *Support person #1* |
|  | Carers knowledge | *“All family members, carers knowing what the signs look like, when to escalate, when to go to the doctor, when to go to hospital.”* *Physiotherapist #2* |
|  | Target the patients  (programme focus) | *“I think you should target the patients. I think the carer is coming along as support and helping the patient to go through the journey, for them to understand. So, I don’t think. You might have carer support as well in terms of carer stress or whatever. They can go to the carer side, whatever it is. But I think, coming back down to the core of it, then the carers understand how a person with delirium is functioning and what they need to pay attention to. Hmm, personally, I don't think we need another one for the carer because I feel like then we are going down a different path.”* *Physiotherapist #2* |
|  | Target the patients  (programme focus) | *“If a carer is a stressed, has burden, then they should be seeking support. You should be giving them this pamphlet, want talk to carer, say, or going–whatever it is. But if you're talking about the rehab being for the patient itself, then I think the support person is coming along the journey to help them understand and to go through the programme with them. Humm.”* *Physiotherapist #1* |
|  | Carers module | *“I think a carers module on delirium and what it is and what it looks like and what the discharge, you know, journey will look like to getting back and what's normal and what's not normal.”* *Exercise physiologist #1* |
|  | Any available support | *“Yeah, carers are gonna be, yeah, if they've got one–or it doesn't necessarily need to be a carer per se, but just a support person, yeah, yeah–to help them keep on track and be accountable.”* *Occupational therapist #2* |
|  | Supporting carers  Making carer support available | *“I think their own booklet or session would be good. Well, again the, the phone call, the once-a-week phone call, if they could have a chat maybe for 5 minutes in that phone call, if they've got any issues, you know “I'm helping Dad do this, but I'm coming across this. Venting and if you can vent if makes you feel much better.”* *Support person #3* |
|  | Carer delirium experience | *“Because you're almost pulled out of your current world and you are thrown into the world of the hospital, and your loved one–and they’ve got no idea what's going on, and you've barely got any idea what's going on, and you've got all these nurses and all these people–big team, big care team–and just trying to navigate is completely and utterly overwhelming for the carer itself, let alone for the person that it's about. And then you're walking into their world of delirium, delusion, you name it.”* *Support person #1* |
|  | Carer stress | *“I think so, because we do get a lot of family members, whoever's providing that support or care, voicing carer stress. So, I think it is–it is something that they would be seeking to a certain degree, like if they are worried about their loved ones, particularly an acute delirium can be really distressing for the family members and the carers to–to witness. So, I think, yeah, and almost like enrolling them also and having a resource also for them to refer to–it's not a bad idea, yeah.”* *Occupational therapist #1* |
|  | Carer education | *“And then, you know, the carer would need the vessel, which is the education, to know what needs to be changed, know who to talk to, or maybe to tap in with support to get new services so that he is better, or whatever it is.”* *Physiotherapist #1* |
|  | Carer education | *“Like actually participate, yeah, I think a carers module on delirium and what it is and what it looks like and what the discharge, you know, journey will look like to getting back and what's normal and what's not normal. With delirium because I think a lot of carers aren't familiar with the presentation and what it, what it does look like. Hmm, yeah, that would be a good like info session.”* *Exercise physiologist #2* |
|  | Carer module | *“Nobody really could tell me what was going on. “Well, your mother's got delirium and–and–and it might–” I was still trying to work out what the delirium means and what that means. So, you really need to do a carer module as well.” Support person #1* |
|  | Carers are forgotten | *“You really have to target the patient and the family either together or individually and have the flexibility to be able to do both or either and certainly, carers are generally the forgotten.”* *Support person #1.* |
| Be a trusted partner | Staff awareness of the programme | *“I think that screening and referrals from hospital will be really important, people to be aware of the programme for the entire. So, you know, planning appropriate referrals. so having it be visual in the hospital.”* *Geriatrician* |
|  | Staff awareness of the programme | *“A lot of discharge is allied health driven. So, if there is sort of allied health from the hospital, hmm, sort of very much aware of the programme, know how to refer slash recruit, then that's gonna be your best bet, cause I guess from our end, we very much, hmm, on discharge plan pathways are very in-house, like SA Health, like downstream rehab sort of pathways that are our own. We don't tend to go into sort of like the private space all that much. So, the more relevant you are in our system conversation maps of–of discharge pathways, the more.”* *Occupational therapist #1* |
|  | Staff awareness of the programme | *“Sort of how to make that referral pathway pretty seamless, because we're all time poor, and not everyone has a discharge coordinator slash planner. So, it's sort of–one patient might have the OT do the referral, the next patient might have the physio or the TL, or so. It's all sort of like a, yeah, team model. Should be easier–the easiest way from a process perspective, the better.”* *Occupational therapist #3* |
|  | Involving health professionals | *“I think when you roll it out, you're gonna have to spread it everywhere. Talk to every ward, because there's gonna be delirium in every–delirium on every ward. Hmm, involve them, the managers. Hmm, put it on the weekly meeting schedules that they all have.” Nurse* |
|  | Role of allied health professionals | *“I think really target allied health as well, cause allied health have a significant role in discharge planning, and this is sort of like a discharge pathway.”* *Occupational therapist #3* |
|  | Tapping into hospital system | *“Is there a box you can put in your discharge planning summary to have–“Have you thought about HALT? Have you had delirium?” Make that a visual thing for every person who–through.”* *Nurse* |
|  | Flagging the programme | *“Is there any way to flag in the EMR? Because I know the medical coders go through and they're like, “delirium, right, OK, this code to it,” hmm, anyway, but that just to automatically have a text message or a letter to the patient saying, “you have had delirium, there is this education programme for you to participate in.”* *Nurse* |
|  | GP awareness | *“Do you send a summary to their GP? That could be where you capture it. Look, we identify them as needing nutrition. Better raise awareness for the GP [inaudible], “Ohh, we can refer him here.”* *Nurse* |
|  | Building rapport | *“I think that's the biggest way that you create people engaged, is that phone call, that weekly check-in, when you slowly build the rapport with them. “Oh hi, I wanted to chat with you.”* *Nurse* |
|  | Building connection | *“I think whoever you have on the other side of that phone call is gonna be your key person, alongside their carers, to help motivate them. So, I wonder whether there are any particular questions you're asking during that phone call to keep it motivating and keep it focused to them.”* *Occupational therapist #1* |
| Overcoming barriers to optimise engagement | Potential barriers  motivation | *“I think motivation will be your biggest challenge. Particularly for people that live out this way who aren't, yeah, the most proactive in a lot of ways, and have, like, entrenched habits that aren't helpful for their health.”* *Occupational therapist #1* |
|  | Motivation | *“The biggest challenges, I think, are just going to be like people not wanting to do it. You know, they-they may-may be in hospital for 2-3 months, they get home and then it’s like, “oh no, I’ve gotta do this kind of study or-or research or reading or whatever they have to do,” and they might just want to relax.”* *Exercise physiologist #1* |
|  | Motivation | *“I think my only concern would be the-the self-motivating and like, the self-driven factor. I think it's going to have a lot of barriers. I think about a lot of the older people that we get in, who their busy day is like one appointment, and they can't really tolerate a lot of other things, so adding kind of this on being a self-driven mode might just fall into the “too hard” basket.”* *Occupational therapist #2* |
|  | Motivation | *“Yeah, when it comes back to that mode of delivery and how much support they need to actually engage in the programme. Some people, I think [inaudible], self-motivated [inaudible], I don’t’ know, they just wouldn't do it [inaudible].”* *Occupational therapist #3* |
|  | Self-driven | *“Since it is a self-driven thing, there’ll be a lot of people dropping out.” Nurse* |
|  | Lifestyles | *“A lot of NALHN population, who aren't overly proactive and live the healthiest lifestyles, have a lot of social barriers against that behavioural change and that appetite to learn. A lot of, you know, illiteracy, a lot of, so that might be a challenge.”* *Occupational therapist #3* |
|  | People difference | *“Every older people are different, hmmm, and I think then you need to identify what mode is better for them. It could be a one-on-one, it could be hearing.”* *Physiotherapist #1* |
|  | Engagement | *“I think that the biggest challenge is gonna be those phone calls and getting them as engaged as possible throughout.”* *Occupational therapist #2* |
|  | Capacity | *“Because if you know that you don't have the capacity, and you're going to get very complex people coming on, and the mode of delivery can be challenging.”* *Physiotherapist #1* |
|  | Mode of delivery | *“It's hard. The best way would be face to face. The second best way would be digital face to face and the third one being your phone call, and then obviously nothing for me, so generally, I think phone-phone call was just gonna have to be, and that's fine. I think if there's other ways to tap into a face to face or that Tele, then great.”* *Occupational therapist #1* |
|  | Complex need | *“The other thing I wanted to say was that the success around having a visual, someone visually impaired, when you are no longer having someone to actually see and interact with.”* *Occupational therapist #2* |
|  | Readiness for change | *“There are things that, you're probably not gonna get them to do because it's inherent in their behaviour from their normal resistance and that probably, it will be more with men than women.”* *Support person #2* |
|  | Readiness for change | *“And a lot of old people get very, very stubborn. So, it's presenting it to them in a way that they want to do it.”* *Support person #3* |
|  | Cognitive capacity | *“I'm just thinking from a cognitive perspective to be able to function at home, to be able to do a delirium programme, and I would think that they would be pretty good if they can do that and have the cognitive function.”* *Physiotherapist #4* |
|  | Literacy | *“Then it could be a simple problem about “what is this medication” or like “I don't know why I'm taking it” and then another person could have literacy skills to understand exactly what you're talking about, the other person just maybe has to open a packet and they're struggling with it.”* *Physiotherapist #5* |
|  | Complex need | *“I think the literacy is very important because I would say not a lot of people will pick up this and actually understanding it. A lot of the older generation could be, you need to talk to them; they will tell you I can't see, it’s visually challenging and it is too much. I think, you know, paper-based is good, but then I feel like then you kind of have this target audience, but then you forgot about the other challenging audience that may not be able to read that print and also understanding and following through.”* *Physiotherapist #4* |
|  | Lack of carer | *“Ideally, there would be a carer with them to do the programme, bit more of a challenging when they don't have a support person.”* *Geriatrician* |
|  | Living alone | *“I think, yeah, for people that are living on their own, they’re gonna be not very motivated.”* *Physiotherapist #2* |
|  | Living alone | *“Because once they are home alone, I think you’re right, they probably not engaged.” Nurse* |
|  | Interactive mode | *“Because if you're giving these and it's not overly interactive because it's just printed–well, then they can read it, but what do I do with this information? Where is it? If it's interactive on the internet–or I know you need to cater for a broad population, which is also another challenge within itself–and the interactive thing on the computer may be a little bit more involved, with games or different things along the way.”* *Support person #1* |
|  | Engagement | *“Is that engaging enough? because some people might like to sign up to it, and then after the first few days or week or whatever, “it's too hard, it’s too much,” particularly depending on how cognitively they're functioning after the delirium experience.”* *Support person #1.* |
|  | Engagement | *“Especially if they are doing the long, like all the modules, and it's going to take six months. I think it'd be hard for people to really engage”* Exercise *physiologist #1* |
|  | Solution for the challenges  Information on recurrence | *“Present it to them as something that is going to be worth-while. “Did you like having delirium?” “No”. “Do you want it again?” “No”. “Do you think you're going to, there is a possibility you will end up in hospital again”. “Yes”. “Well, do you want delirium again?” “No”. Well, pretty self-explanatory to me, I think it has to be really really-really-really explained to them.”* *Support person #2* |
|  | Information on trajectory | *“I think it's time critical, and the sooner you've–you've got the person who and the carer who understands what's going on in the trajectory would be a lot easier than trying to walk through the mist and trying to walk through the maze of, “Where are we? What are we doing?”* *Support person #1* |
|  | Expectation | *” The screening to have that kind of realistic talk of: how long do you think you can withstand going through this course? You know, especially if you do identify someone needs all 8 sessions, but you have to help them pick through realistically.”* *Occupational therapist #1* |
|  | Information on recurrence | *“I think that will be a big draw card for people, if they're [talk overlap] [inaudible], “I've been scared by it, I don't want it again.”* *Nurse* |
|  | Information on recurrence | *“They don't wanna get it again. Dad didn't want to get it again, so raising awareness will be a motivating thing.” Support person #3* |
|  | Information on need | *“At the same time if you give them the results of whatever test you do for each module. Because they might not know that they are malnourished, and they might not know their body [inaudible] or something. So, you saying, “Ohh, you did score quite low in this area. I think this would be good,” might make them–it could be, “Oh great, I’ll do that.”* *Exercise physiologist #1* |
|  | Meaningful phone calls | *“How are you making these phone calls meaningful to them when they're going through that module? If the conversation is going to go for 5 minutes versus 40 minutes to an hour, how much are you probing them to get the most out of that phone call, as opposed to just a check-in? So, when we pick up the phone and say, you know, “How are you going with the first module?” They say “Good.” What else are we trying to get out of this?* *Occupational therapist #1* |
|  | Stay relevant | *“Almost like you need to stay relevant for them–like, like–like a consistent presence, but how to achieve that within sort of the limitations–resources and funding and bodies, staffing–is the tricky part.”* *Occupational therapist #1* |
|  | Goal setting | *“I think just goal setting would be probably the biggest one. Just, hmmm, getting them to create their own goals around each topic.”* *Exercise physiologist #1* |
|  | Meeting reminder | *“Maybe just like saying, I’m gonna ring you Tuesday; if you can, you know, have a look at this module between now and Tuesday, and then we can really discuss it. Just giving, yeah, a bit of a time frame of when they might need to”* *Physiotherapist #1* |
|  | Flexibility | *“Hmm, I think making it flexible, so if someone does really need to hospital for something, or–or, you know, you can pause it for, you know, a couple of weeks or something, or if, you know, you've got other stuff on, you can.”* *Exercise physiologist #1* |
|  | Starting the programme | *“By two weeks, the delirium would be gone and, that, but if it was four weeks after discharge, you had no, no real hope of engaging him in the programme, I would say. Because I just think four weeks after discharge, a lot of them won't even remember.”* *Support person #2* |
| Comprehensive content and easy to read materials | Addressing need | *“I think it covers all the basics overall that we discussed in the first meeting.”* *Physiotherapist #5* |
|  | Comprehensive | *“I think they're good, I can't think of anything else that you could possibly cover.”* *Support person #3* |
|  | Comprehensive | *“I think it’s pretty comprehensive.”* *Exercise physiologist #1* |
|  | Content coverage | *“A lot to take in.”* *Person who experienced delirium.* |
|  | Interesting programme | *“This would’ve really appealed to Dad, how you’ve put this together.”* *Support person #2* |
|  | Module stories | *“I like the story pretty much, because a bit further on in pages, he gets better.”* *Person who experienced delirium.* |
|  | Module stories | *“I think–I don't know how many modules are tied up with that type of thing, but if there's any–if that's the only module in that part of the area, I probably want that module, because if I'm reading stuff and trying to–at the age of 83, coming up in December, I’m 83–hmm, yeah, I won't be overly interested. I'll be interested in that module.”* *Person who experienced delirium.* |
|  | Module stories | *“I think the stories are good. That's a good way to keep them connected with, with doing this stuff.”* *Support person #3* |
|  | Easy to read | *“Very, very easy to read.* *I did li- like the stories.”* *Support person #3* |
|  | Booklet font size | *“I think they're a, they're a good size for elderly people, and I wouldn’t have them any bigger, and I wouldn’t have them any smaller. The font it good. The font’s easy to read.”* *Support person #3* |
|  | Booklet arrangements | *“Font size is great. I love the colour; I love the pictures. It's not too wordy.” Nurse* |
|  | Booklet size | *“I think, yeah, the print material being the right- the right size. I think that's much easier to read the- the A4 other than A5.”* *Occupational therapist #2* |
|  | Booklet colour | *“I like the colours, and I like the font on the A4 version. absolutely, that sign posted. I liked that story.”* *Geriatrician* |
|  | Space for reflection | *“I like the way they interact with it and write what they want, and then that's cool.”* *Physiotherapist #1* |
|  | Rearranging the contents | *“Actually, putting that page somewhere towards the front really would be good. I think that, that's really, really important page because when you tell someone they've got del- delirium, they immediately think they have dementia.”* *Support person #2* |
|  | Printed material | “Paper-based is good as well. I think the printed material is good.” *Geriatrician* |
|  | Bit by bit | “I do like the idea, if you were mailing these out. Humm, you know, and maybe if they weren’t all bound out in one giant booklet but bit by bit.” *Occupational therapist #1* |
|  | Need based recommendation | *“I think it's a good approach; I think the wording and how you present it will be very important.”* *Geriatrician* |
|  | Addressing need | “I mean, I-I-I want help if I can, and that's what the programme is about.” *Person who experienced delirium.* |
|  | Participation | “Certainly, from what I've read, yes.” *Person who experienced delirium.* |
|  | Addressing need | “I think that's really good because Dad’s nutrition wasn't what, was not always good.” *Support person #3* |
|  | Addressing need | “Well, I can tell you now that they get very, they can get very sneaky with their medication.” *Support person #2* |
|  | Interesting modules | “I really like this one: what is delirium?” *Physiotherapist #5* |
|  | Recommending the programme | “Without a doubt.” *Support person #2* |
|  | Programme importance | “So, I think what you guys are doing is really, really important because as you said, up to 50% of elderly people may just be suffering with delirium.” *Support person #2* |
|  | Programme importance | “I haven't got anything to add. I would just like to commend you guys for trying to do something like this because, it would have been nice.” *Support person #3* |
|  | Success of the programme | *“I think it's going to be pretty successful just judging what you have. I think it's so unique.”* *Exercise physiologist #1* |
|  | Success of the programme | *“If you got the right person and the right support network and the right appetite to learn and the right appetite to make changes, then yes, it will be successful.”* *Occupational therapist #1* |
